# Effects of Systemic and Skeletal Muscle–Specific Overexpression of Piezo1

**DOI:** 10.24546/0100495773

**Published:** 2025-05-01

**Authors:** TOMOYA INOUE, TOMOKO NISHIGAKI, YU HIRATA, KAZUHIRO NOMURA, KENJI SUGAWARA, WATARU OGAWA

**Affiliations:** 1 Department of Diabetes and Endocrinology, Department of Internal Medicine, Kobe University Graduate School of Medicine, Kobe, Japan; 2 Department of Nutrition and Metabolism, Institute of Biomedical Sciences, Tokushima University Graduate School, Tokushima, Japan

**Keywords:** Piezo1, Overexpression, Immobilization, Muscle atrophy, Hemolytic anemia

## Abstract

Piezo1, a mechanosensitive ion channel that opens in response to mechanical stimuli, is widely expressed among mammalian cell types, and regulates a diverse range of physiological processes. Although evidence has suggested potential clinical benefit of Piezo1 activation for various conditions, the safety and efficacy of such activation in living animals have remained unclear. To investigate the therapeutic potential of Piezo1 activation, we here generated genetically modified mouse models in which Piezo1 is overexpressed either specifically in skeletal muscle or systemically in response to tamoxifen treatment in adult animals. Cast immobilization induced a reduction in both muscle mass and the abundance of *Piezo1* mRNA in skeletal muscle of the affected limbs in control mice. Overexpression of Piezo1 in skeletal muscle prevented the immobilization-induced reduction both in soleus muscle mass and in the corresponding cross-sectional area of myofibers, suggesting the potential benefit of Piezo1 activation for prevention of immobilization-induced muscle atrophy. Furthermore, mice with systemic overexpression of Piezo1 showed no apparent abnormalities in growth or general activity. Red blood cells from these mice manifested slight resistance to hypoosmolarity-induced hemolysis, and the animals did not develop apparent hemolytic anemia. Our findings demonstrate promising efficacy and safety of Piezo1 activation in living animals and thereby highlight the therapeutic potential of targeting the Piezo1 signaling pathway.

## INTRODUCTION

Piezo1 is a mechanosensitive ion channel that opens in response to mechanical stimuli [1]. Whereas its closely related isoform Piezo 2 is expressed exclusively in Merkel cells of skin and contributes to tactile sensation, Piezo1 is widely expressed among mammalian cell types [2, 3] and regulates a wide range of physiological processes including muscle regeneration [4], maintenance of bone mass [5], adipocyte differentiation [6], and recovery from hepatic inflammation [7]. Moreover, we recently showed that limb immobility results in downregulation of Piezo1 expression in limb skeletal muscle, leading to muscle atrophy through enhanced protein catabolism [8]. These findings suggest a potential clinical benefit of Piezo1 activation for various conditions including osteopenia, obesity-related metabolic disorders, hepatitis, and disuse-related muscle atrophy. However, screening of ~3.25 million compounds identified only a single agent, Yoda1, as a Piezo1 activator [9], the therapeutic application of which in humans remains challenging because of its toxicity. The relevance of Piezo1 activation as a therapeutic approach thus remains uncertain.

Validation of the Piezo1 pathway as a suitable target for drug development will require evaluation of the safety of Piezo1 activation. In this context, individuals with gain-of-function variants of the Piezo1 gene have been identified, some of whom manifest no apparent pathological conditions but have shown enhanced athletic performance, which may be attributable in part to improved tendon function [10]. However, other such individuals have been found to develop conditions such as hemolytic anemia [11, 12], splenomegaly [13], and lymphedema [14]. These latter findings raise concerns about the potential safety of Piezo1-activating drugs, although whether activation of the Piezo1 pathway in adulthood would have the same effects as congenital activation remains unknown.

We have now generated mice that overexpress Piezo1 from the *Rosa26* locus in a Cre-dependent manner. These mice were crossed with mice that express Cre recombinase in a tamoxifen-inducible manner either selectively in skeletal muscle or systemically. With the use of these models, we have investigated the potential efficacy and safety of Piezo1 activation in the living body.

## MATERIALS AND METHODS

### Materials

#### Animal experiments

All animal experiments were performed according to the guidelines of and were approved by the Animal Experimentation Committee of Kobe University Graduate School of Medicine (approval no. P221201-R1). For generation of conditional Piezo1 knock-in mice at the *Rosa26* locus (R26-Piezo1 KI mice), a homologous recombination vector for *Rosa26* that contains the CAG promoter, a chloramphenicol acetyltransferase (CAT) gene flanked by loxP sites and a neomycin resistance (neo) gene flanked by FRT sites as a STOP cassette, and mouse *Piezo1* cDNA was constructed (Fig. S1A). The vector was introduced into mouse embryonic stem cells by electroporation, and cell clones having undergone recombination were selected with G418. The selected clones were injected into mouse blastocysts, and the resultant mice were crossed with CAG-FLPe mice [15] to remove the neo gene (Fig. S1B). HSA-Cre-ERT2 mice, which express Cre specifically in muscle fiber cells in response to tamoxifen treatment, were as previously described [16], and R26-Cre-ERT2 mice, which express Cre systemically in a tamoxifen-inducible manner, were obtained from the Jackson Laboratory (Bar Harbor, ME, USA). All mice were backcrossed more than six times with C57BL/6J mice. R26-Piezo1 KI mice were crossed with HSA-Cre-ERT2 or R26-Cre-ERT2 mice to generate mice that overexpress Piezo1 in skeletal muscle (mOE mice) or systemically (sOE mice), respectively, in response to tamoxifen treatment (Fig. S1B). The sequences of polymerase chain reaction (PCR) primers for genotyping are listed in Table SI, and representative results of such genotyping are shown in Fig. S1C. Bilateral cast immobilization of mouse hind limbs with the use of plastic tubes and adhesive tape was performed as previously described [8]. R26-Piezo1 KI mice were used as the control mice. Unless otherwise specified, 10-week-old male mice were used for all experiments.

### Methods

#### RT-qPCR analysis

Reverse transcription (RT) and quantitative PCR (qPCR) analysis was performed essentially as described previously [17] with the use of an ABI StepOne Plus Real-Time PCR system (Applied Biosystems, Waltham, MA, USA). Total RNA was extracted with the use of specific kits based on tissue type. Liver, lung, kidney, spleen, and pancreas were processed with a RNeasy Mini Kit, skeletal muscle and heart with a RNeasy Fibrous Tissue Mini Kit, and white adipose tissue (WAT) and bone marrow with a RNeasy Lipid Tissue Mini Kit (Qiagen, Hilden, Germany). Data were normalized by the amount of *Rplp0* mRNA. The sequences of PCR primers are provided in Table SII.

#### Histological analysis

Sections of skeletal muscle were stained with hematoxylin-eosin. Images were randomly captured with a BZ-X700 microscope (Keyence, Osaka, Japan), and the cross-sectional area of myofibers was measured with the use of BZ-X700 Analyzer (Keyence).

#### Hematology

Blood samples were obtained from the tail vein and promptly treated with heparin. The level of hemoglobin was determined with a Celltac α MEK-6550 analyzer (Nihon Kohden, Tokyo, Japan). The morphology of red blood cells (RBCs) was observed with a BZ-X700 microscope (Keyence Corporation, Osaka, Japan).

#### Osmotic fragility test

Blood was diluted 1:50 with normal saline (154 mM NaCl), and portions (10 μl) of the diluted blood were transferred to the wells of a U-bottom 96-well plate. Solutions of different tonicities were prepared by mixing normal saline (100%) with distilled water (0%), and 250 μl of each solution were added to the diluted blood samples and incubated for 5 min at room temperature. The plate was then centrifuged, and 100 μl of the supernatant in each well were transferred to the wells of a flat-bottom 96-well plate for measurement of absorbance at 540 nm with an iMark Microplate Reader (Bio-Rad, San Francisco, CA, USA).

#### Statistical analysis

Quantitative data are presented as means ± SEM, unless indicated otherwise, and were analyzed by the two-tailed unpaired Student’s *t* test or by two-way analysis of variance (ANOVA) with Tukey’s multiple comparison test. All statistical analysis was performed with GraphPad Prism version 9.0 (GraphPad Software, San Diego, CA, USA). A *P* value of <0.05 was considered statistically significant.

## RESULTS

### 1. Acute expression of Piezo1 in skeletal muscle attenuates immobilization-induced muscle atrophy

**Fig. 1:**
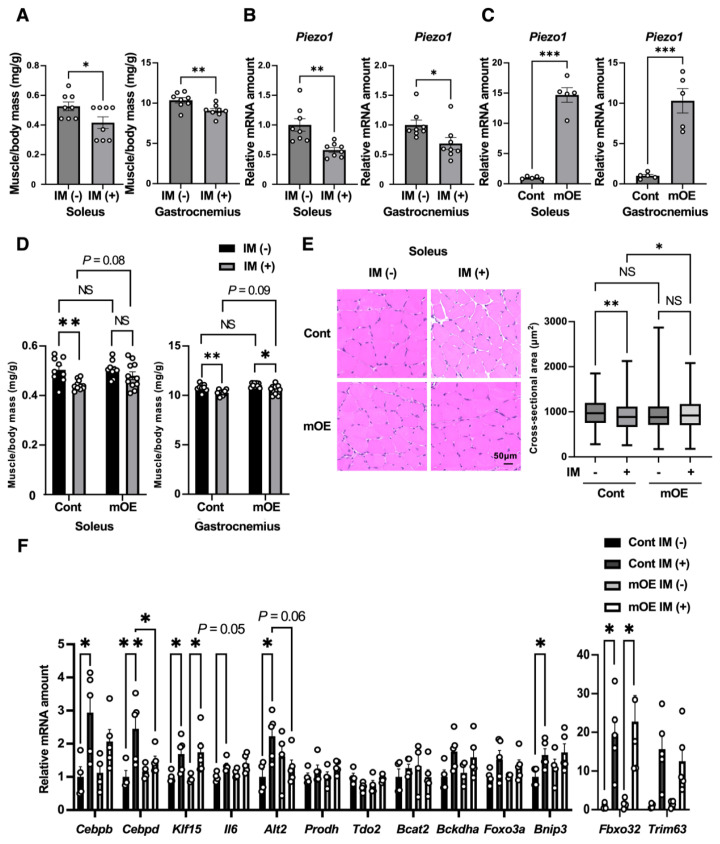
Effects of acute overexpression of Piezo1 in mouse skeletal muscle during limb immobilization (A and B) Soleus and gastrocnemius muscle mass (A) and relative *Piezo1* mRNA abundance in the muscle tissue (B) at 3 days after bilateral immobilization (IM) of hind limbs with a plastic cast. Nonimmobilized mice were examined as a control. Data are means ± SEM (*n* = 8 mice). (C) RT-qPCR analysis of relative *Piezo1* mRNA abundance in soleus and gastrocnemius of control (Cont) mice and mice that overexpress Piezo1 in skeletal muscle in a tamoxifen-inducible manner (mOE mice). The analysis was performed 7 days after the second of two consecutive daily intraperitoneal injections of tamoxifen (0.3 mg/body). Data are means ± SEM (*n* = 5 mice). (D–F) Control and mOE mice were treated with tamoxifen on days −1 and 0 and then subjected to bilateral cast immobilization of hind limbs from 4 to 7 days before analysis. (D) Soleus and gastrocnemius muscle mass. Data are means ± SEM (*n* = 9 mice [IM (−)], *n* = 11 mice [IM (+)]). (E) Hematoxylin-eosin staining of soleus and quantification of myofiber cross-sectional area. Scale bar, 50 μm. Quantitative data are presented as box plots, with the boxes indicating the median and quartile values and the whiskers indicating the minimum and maximum values (*n* = 4 mice [IM (−)], *n* = 5 mice [IM (+)]). (F) Atrophy-related gene expression in soleus. Data are means ± SEM (*n* = 4 mice [IM (−)], *n* = 5 mice [IM (+)]). *P < 0.05, **P < 0.01, ***P < 0.001 by the two-tailed unpaired Student’s *t* test (A–C) or two-way ANOVA and Tukey’s post hoc test (D–F). NS, not significant.

Bilateral cast immobilization of the hind limbs of mice for 3 days resulted in a reduction in the mass of the soleus and gastrocnemius muscles (Fig. 1A) that was accompanied by downregulation of *Piezo1* mRNA abundance (Fig. 1B), consistent with our previous findings [8]. Intraperitoneal administration of tamoxifen on two consecutive days in mice that overexpress Piezo1 specifically in skeletal muscle in a tamoxifen-inducible manner (mOE mice) resulted in a 10- to 15-fold increase in the amount of *Piezo1* mRNA in soleus and gastrocnemius at 7 days after the second administration of the drug (Fig. 1C).

We next examined the effects of Piezo1 overexpression on immobilization-induced muscle atrophy. Whereas cast immobilization resulted in a significant 12.8% reduction in soleus muscle mass in control (Cont) mice, tamoxifen-treated mOE mice did not show a significant difference in soleus mass between the nonimmobilized and immobilized conditions, with the reduction in muscle mass after immobilization being limited to 5.8% (Fig. 1D). In contrast, gastrocnemius muscle mass was significantly reduced by cast immobilization in both tamoxifen-treated control and mOE mice (Fig. 1D), with the extent of the reduction being slightly smaller in mOE mice (4.2%) compared with control mice (4.9%). Histological analysis revealed that the immobilization-induced reduction in the cross-sectional area of soleus muscle fibers apparent in control mice was abolished in tamoxifen-treated mOE mice (Fig. 1E). Collectively, these findings indicated that Piezo1 overexpression in skeletal muscle mitigates immobilization-induced atrophy in soleus muscle.

Whereas limb immobilization upregulated the expression of various genes related to protein catabolism in skeletal muscle of control mice, as reported in our previous study [8], overexpression of Piezo1 significantly suppressed the immobilization-induced expression of *Cebpd* and tended to suppress that of *Cebpb* and *Alt2* in soleus (Fig. 1F).

### 2. Acute systemic overexpression of Piezo1

**Fig. 2:**
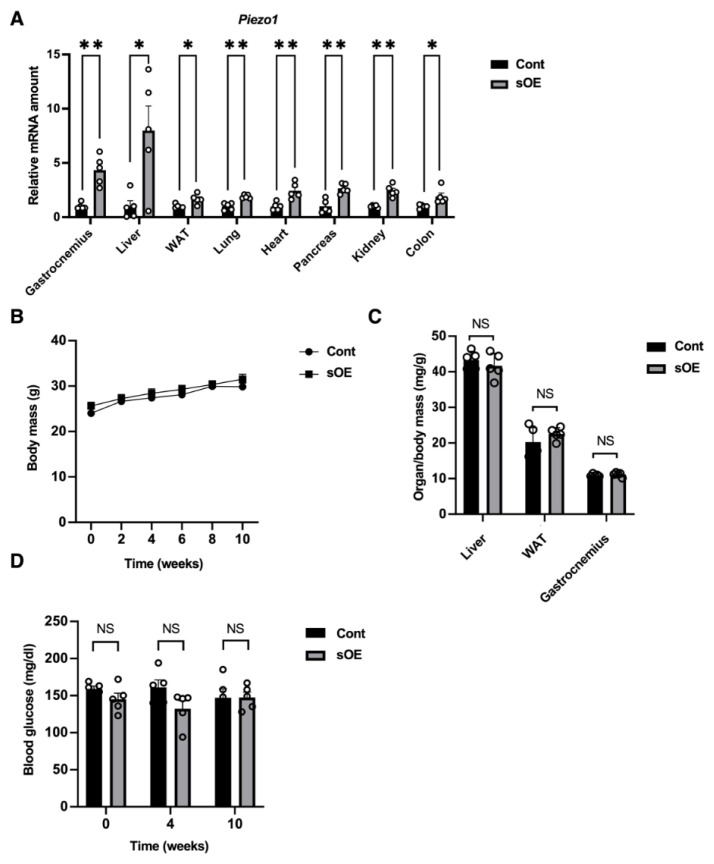
Phenotype of systemic Piezo1-overexpressing mice (A) RT-qPCR analysis of relative *Piezo1* mRNA abundance in various organs of control (Cont) mice and mice that overexpress Piezo1 systemically in a tamoxifen-inducible manner (sOE mice). The analysis was performed 10 weeks after intraperitoneal injection of tamoxifen (0.3 mg/body) on two consecutive days. Data are means ± SEM (*n* = 5 mice). (B) Body mass of control mice and sOE mice at the indicated times after tamoxifen administration as in (A). Data are means ± SEM (*n* = 5 mice). (C) Mass of the liver, WAT, and gastrocnemius muscle of control and sOE mice at 10 weeks after tamoxifen administration. Data are means ± SEM (*n* = 5 mice). (D) Blood glucose concentration of control mice and sOE mice at the indicated times after tamoxifen administration. Data are means ± SEM (*n* = 5 mice). *P < 0.05, **P < 0.01 by the two-tailed unpaired Student’s *t* test. NS, not significant.

We next investigated the effects of systemic overexpression of Piezo1. Tamoxifen administration in mice that overexpress Piezo1 systemically in a tamoxifen-inducible manner (sOE mice) resulted in a marked increase in the abundance of *Piezo1* mRNA in various tissues, including skeletal muscle, liver, WAT, lung, heart, pancreas, kidney, and colon (Fig. 2A). The tamoxifen-treated sOE mice showed no apparent changes in general appearance or daily activity. Moreover, the age-dependent gain in body mass (Fig. 2B), the mass of major organs including the liver, WAT, and skeletal muscle (Fig. 2C), and blood glucose levels (Fig. 2D) were also similar between tamoxifen-treated sOE and control mice. These results suggested that systemic Piezo1 overexpression does not induce serious health disorders in mice.

### 3. Evaluation of hemolysis in mice with systemic overexpression of Piezo1

**Fig. 3:**
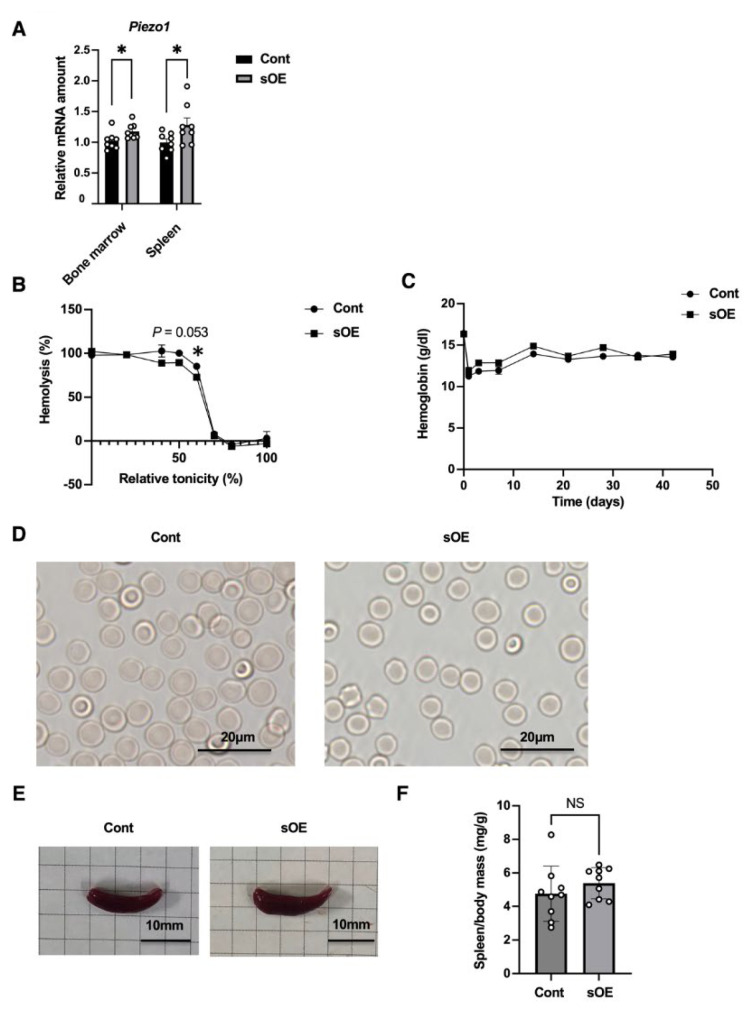
Evaluation for hemolytic anemia and splenomegaly in mice with acute systemic overexpression of Piezo1 (A) RT-qPCR analysis of relative *Piezo1* mRNA abundance in bone marrow and spleen of control (Cont) and sOE mice at 6 weeks after intraperitoneal injection of tamoxifen (0.3 mg/body) on two consecutive days. Data are means ± SEM (*n* = 8 mice). (B) Osmotic fragility test for blood obtained from control and sOE mice at 6 weeks after tamoxifen administration as in (A). Data are means ± SEM (*n* = 6 independent experiments). (C) Hemoglobin levels of control and sOE mice at the indicated times after tamoxifen administration. Data are means ± SEM (*n* = 9 mice). (D) Morphology of RBCs obtained from control and sOE mice at 6 weeks after tamoxifen administration. Scale bars, 20 μm. (E and F) Appearance (E) and mass (F) of the spleen from control and sOE mice at 6 weeks after tamoxifen administration. Scale bars, 10 mm. Quantitative data are means ± SEM (*n* = 9 mice). *P < 0.05 by the two-tailed unpaired Student’s *t* test. NS, not significant.

Given that individuals with certain gain-of-function variants of Piezo1 develop hemolytic anemia [18], we next evaluated sOE mice for this condition. The lifespan of RBCs in C57BL/6 mice is ~35 days [19], and we therefore assessed the effects of Piezo1 overexpression at 6 weeks after tamoxifen treatment, a time point that would be expected to allow for sufficient RBC turnover. At this time, the abundance of *Piezo1* mRNA in hematopoiesis-related organs including bone marrow and spleen was moderately but significantly higher in sOE mice than in control mice (Fig. 3A).

Mice harboring a gain-of-function variant of Piezo1 specifically in erythroid-lineage cells were also previously found to develop hemolytic anemia and consequent splenomegaly, and their RBCs showed increased resistance to hypotonic stress [18]. Whereas hemolysis of RBCs obtained from control mice increased as the osmotic pressure decreased, hypoosmolarity-induced hemolysis was attenuated for RBCs from systemic Piezo1-overexpressing mice (Fig. 3B), an observation similar to that for the mice harboring the gain-of-function Piezo1 variant.

Tamoxifen treatment induced a transient decrease in hemoglobin levels in both control and sOE mice, after which the levels remained similar between the two genotypes throughout the observation period (Fig. 3C). In addition, RBCs from tamoxifen-treated sOE mice showed no apparent morphological abnormalities (Fig. 3D), in contrast to those from mice harboring the gain-of-function Piezo1 variant in erythroid-lineage cells, which manifested stomatocytosis [18]. Furthermore, the appearance (Fig. 3E) and mass (Fig. 3F) of the spleen were similar between tamoxifen-treated control and sOE mice, providing further evidence against the development of hemolytic anemia in the latter animals.

## DISCUSSION

We have here shown that overexpression of Piezo1 in skeletal muscle of adult mice achieved with the use of tamoxifen-inducible gene recombination prevents muscle atrophy induced by limb immobilization. This finding not only supports the notion that impaired Piezo1 signaling is a key factor in immobility-induced muscle atrophy but also highlights the potential of Piezo1 as a therapeutic target for this condition. The amelioration of muscle atrophy was observed in soleus muscle, which consists predominantly of slow-twitch fibers, but was less apparent in gastrocnemius, a mixed muscle composed of both slow- and fast-twitch fibers. This suggests that the beneficial effects of Piezo1 activation may differ between muscle types.

Limb immobilization results in the upregulation of various genes related to protein catabolism, likely contributing to the development of muscle atrophy. However, Piezo1 overexpression had minimal effects on the expression of these genes, suggesting that the antiatrophic effect of Piezo1 is achieved through a mechanism independent of the suppression of protein catabolic genes. Given that Piezo1 activates the mechanistic target of rapamycin (mTOR) signaling pathway [20], which contributes to muscle hypertrophy [21], it is possible that this pathway mediates the antiatrophic action of Piezo1 in skeletal muscle.

Mice with systemic overexpression of Piezo1 showed no apparent abnormalities in growth or general activity. Whereas these observations do not exclude potential adverse effects of systemic Piezo1 activation, they are consistent with the fact that individuals harboring certain gain-of-function Piezo1 variants—such as the E756 deletion mutant, which is present in ~18% of individuals of African descent—are generally healthy and show resistance to malaria as well as enhanced athletic performance [10]. RBCs from mice with systemic overexpression of Piezo1 manifested resistance to hypotonic stress, a phenomenon also observed for RBCs of mice genetically engineered to express a gain-of-function Piezo1 variant specifically in erythroid-lineage cells [18]. These findings suggest that systemic overexpression of Piezo1 in sOE mice leads to exaggerated Piezo1 signaling in erythroid-lineage cells. Nonetheless, mice with systemic overexpression of Piezo1 did not develop anemia, morphological changes in RBCs, or splenomegaly, which may reflect differences in the effects of Piezo1 activation as a congenital condition versus during adulthood. Alternatively, they may be simply attributable to differences in the extent of Piezo1 activation in RBCs between the two mouse models.

One limitation of our study is that we were not able to assess the extent of Piezo1 overexpression in RBCs of mice with systemic Piezo1 overexpression. Although we performed immunoblot and flow cytometric analyses, we were unable to detect Piezo1 signals in RBCs of either control or mutant mice. Evaluation of Piezo1 protein abundance is inherently challenging, as highlighted by previous studies that adopted genetic labeling methods to quantify Piezo1 protein levels [22, 23]. A second limitation is that, whereas mice with systemic Piezo1 overexpression appeared generally healthy, detailed assessments of organ function remain to be undertaken.

In conclusion, by generating new mouse models with inducible Piezo1 expression, we have shown that acute overexpression of Piezo1 in skeletal muscle attenuates immobility-induced muscle atrophy, suggesting the therapeutic potential of Piezo1 activation for this condition. Furthermore, we found that systemic Piezo1 overexpression appeared to be relatively safe. Further investigation is needed to provide greater insight into the efficacy and safety of Piezo1 activators for therapeutic applications.

## Supplementary Information

Fig. S1 Generation of *Rosa26-Piezo1* knock-in mice
(A) Design and construction of the *Rosa26* homologous recombination vector. The vector was designed to include a STOP cassette that comprised a chloramphenicol acetyltransferase (CAT) gene flanked by loxP sites and a neomycin resistance (neo) gene flanked by FRT sites and which was inserted between the CAG promoter and the target *Piezo1* gene sequence. Mouse *Piezo1* cDNA (7.6 kbp) and the polyadenylation signal of the bovine growth hormone gene (bGH pA) were amplified by PCR for insertion into the *Rosa26* knock-in vector. (B) Generation of *Rosa26-Piezo1* knock-in (R26-Piezo1 KI) mice. The *Rosa26* homologous recombination vector was introduced into embryonic stem cells by electroporation, and cell clones that had undergone homologous recombination were selected with G418. Selected clones were injected into blastocysts to generate mice. The neo cassette was removed by crossing the resulting mice with CAG-FLPe mice to generate R26-Piezo1 KI mice. Subsequent crossing with Cre mice resulted in removal of the remnant of the STOP cassette by Cre recombinase, thereby allowing Cre-dependent expression of Piezo1. (C) Genotyping of R26-Piezo1 KI mice. Specific primers were designed for PCR amplification. For detection of the wild-type (WT) allele, a forward primer located in the 5′ homology arm (Piezo1-KI [FWD]) and a reverse primer in the 3′ homology arm (Piezo1-KI [REV-1]) generated a 382-bp PCR product. For detection of the knock-in (KI) allele, a reverse primer targeting the Piezo1 knock-in cassette (Piezo1-KI [REV-2]) was used together with Piezo1-KI (FWD) to generate a 522-bp product. Genotyping results are shown for WT, KI/−, and KI/KI genotypes.

**Table SI:** Primer sequences for genotyping

Primer	Sequence (5′→3′)
Piezo1-KI (FWD)	AAGCACGTTTCCGACTTGAG
Piezo1-KI (REV-1)	ACCAGGTTAGCCTTTAAGCC
Piezo1-KI (REV-2)	CCCCAGAATAGAATGACACC
Cre-ERT2 (FWD)	ATTTGCCTGCATTACCGGTC
Cre-ERT2 (REV)	ATCAACGTTTTCTTTTCGGA

**Table SII:** Primer sequences for RT-qPCR analysis

Mouse gene	Forward primer (5′→3′)	Reverse primer (5′→3′)
*Piezo1*	ATCCTGCTGTATGGGCTGAC	AAGGGTAGCGTGTGTGTTCC
*Cebpb*	CGCCTTTAGACCCATGGAAG	GGTAGGGGCTGAAGTCGATG
*Cebpd*	CACGACTCCTGCCATGTACG	AGTTCGTCGTGGCACAGCTC
*Klf15*	ACCGAAATGCTCAGTGGGTTACCTA	GGAACAGAAGGCTTGCGAGTCA
*Il6*	CAAAGCCAGAGTCCTTCAGAG	GCCACTCCTTCTGTGACTCC
*Alt2*	CAGACCCAGACAACATTTACCTG	CGCGGAGTACAAGGGATACTG
*Prodh*	TCATCAGTGCCCGCACCTAC	TGCAGTGAGCTTAATGGCTGAGA
*Tdo2*	TGCTCAAGGTGATAGCTCGGA	AGGAGCTTGAAGATGACCACCA
*Bcat2*	TGGAGTGGAATAACAAGGCTG	GTCTCCACCTTTGTATGCTTTC
*Bckdha*	GCAGCCTATGCTGCCAAGC	GATGGCATAGCCATTGTTCCG
*Foxo3a*	CAGGCTCCTCACTGTATTCAGCTA	CATTGAACATGTCCAGGTCCAA
*Fbxo32*	GCAAACACTGCCACATTCTCTC	CTTGAGGGGAAAGTGAGACG
*Trim63*	GCTGGTGGAAAACATCATTGACAT	CATCGGGTGGCTGCCTTT
*Bnip3*	TTCCACTAGCACCTTCTGATGA	GAACACCGCATTTACAGAACAA
*Rplp0*	GAGGAATCAGATGAGGATATGGGA	AAGCAGGCTGACTTGGTTGC
