# Supplementary figures and images for: Effects of Systemic and Skeletal Muscle–Specific Overexpression of Piezo1
Source: Kobe J Med Sci. 2025 May 1;71(1):E31–40. doi: 10.24546/0100495773 (PMC12455910; doi:10.24546/0100495773)

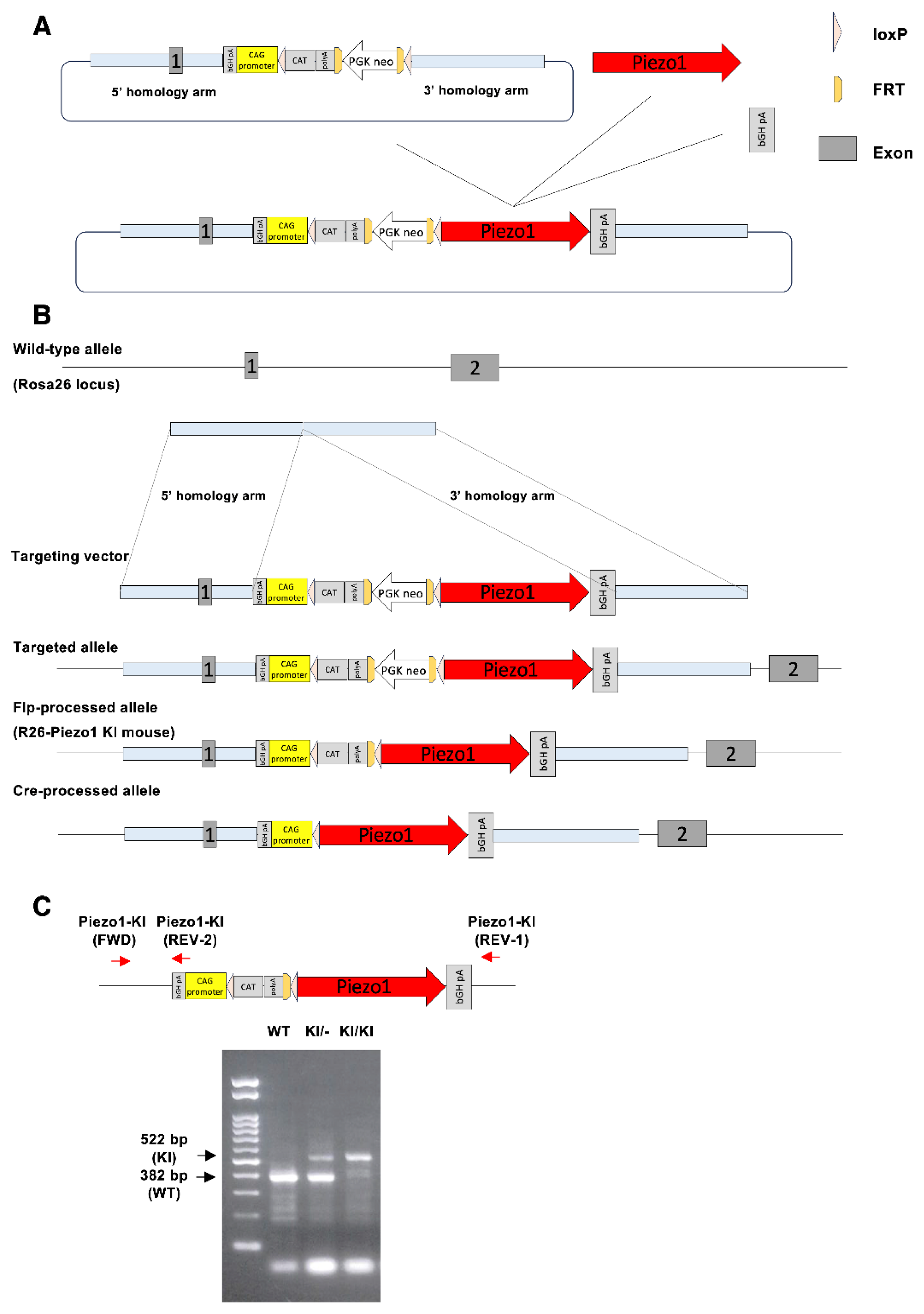

Supplement: Fig. S1 — Generation of Rosa26-Piezo1 knock-in mice (A) Design and construction of the Rosa26 homologous recombination vector. The vector was designed to include a STOP cassette that comprised a chloramphenicol acetyltransferase (CAT) gene flanked by loxP sites and a neomycin resistance (neo) gene flanked by FRT sites and which was inserted between the CAG promoter and the target Piezo1 gene sequence. Mouse Piezo1 cDNA (7.6 kbp) and the polyadenylation signal of the bovine growth hormone gene (bGH pA) were amplified by PCR for insertion into the Rosa26 knock-in vector. (B) Generation of Rosa26-Piezo1 knock-in (R26-Piezo1 KI) mice. The Rosa26 homologous recombination vector was introduced into embryonic stem cells by electroporation, and cell clones that had undergone homologous recombination were selected with G418. Selected clones were injected into blastocysts to generate mice. The neo cassette was removed by crossing the resulting mice with CAG-FLPe mice to generate R26-Piezo1 KI mice. Subsequent crossing with Cre mice resulted in removal of the remnant of the STOP cassette by Cre recombinase, thereby allowing Cre-dependent expression of Piezo1. (C) Genotyping of R26-Piezo1 KI mice. Specific primers were designed for PCR amplification. For detection of the wild-type (WT) allele, a forward primer located in the 5′ homology arm (Piezo1-KI [FWD]) and a reverse primer in the 3′ homology arm (Piezo1-KI [REV-1]) generated a 382-bp PCR product. For detection of the knock-in (KI) allele, a reverse primer targeting the Piezo1 knock-in cassette (Piezo1-KI [REV-2]) was used together with Piezo1-KI (FWD) to generate a 522-bp product. Genotyping results are shown for WT, KI/−, and KI/KI genotypes. [file kobej-71-e31s1.tif]
